# Validation of a Microwave-Assisted Derivatization Gas Chromatography-Mass Spectrometry Method for the Quantification of 2-Hydroxybutyrate in Human Serum as an Early Marker of Diabetes Mellitus

**DOI:** 10.3390/molecules27061889

**Published:** 2022-03-15

**Authors:** María Rodríguez-García, Guillermo Fernández-Varo, Susana Hidalgo, Gabriela Rodríguez, Irene Martínez, Muling Zeng, Eudald Casals, Manuel Morales-Ruiz, Gregori Casals

**Affiliations:** 1 Service of Biochemistry and Molecular Genetics, Hospital Clinic Universitari, Centro de Investigación Biomédica en Red de Enfermedades Hepáticas y Digestivas (CIBERehd), Institut d’Investigacions Biomèdiques August Pi i Sunyer (IDIBAPS), 08036 Barcelona, Spain; mrodriguezg@clinic.cat (M.R.-G.); guillermo.fernandez@ciberehd.org (G.F.-V.); shidalg1@clinic.cat (S.H.); garodrig@clinic.cat (G.R.); imartin1@clinic.cat (I.M.); morales@clinic.cat (M.M.-R.); 2 Department of Biomedicine, University of Barcelona, 08905 Barcelona, Spain; 3 School of Biotechnology and Health Sciences, Wuyi University, Jiangmen 529020, China; mulingzeng@163.com; 4 Commission for the Biochemical Assessment of Hepatic Disease-SEQCML, 08036 Barcelona, Spain

**Keywords:** 2-hydroxybutyrate, microwave-assisted derivatization, *diabetes mellitus*, GC-MS, TMS, human serum

## Abstract

Circulating levels of 2-hydroxybutyrate (2HB) are highly related to glycemic status in different metabolomic studies. According to recent evidence, 2HB is an early biomarker of the future development of dysglycemia and type 2 *diabetes mellitus* and may be causally related to the progression of normal subjects to impaired fasting glucose or insulin resistance. In the present study, we developed and validated a simple, specific and sensitive gas chromatography-mass spectrometry (GC-MS) method specifically intended to quantify serum levels of 2HB. Liquid–liquid extraction with ethyl acetate was followed by 2 min of microwave-assisted derivatization. The method presented acceptable accuracy, precision and recovery, and the limit of quantification was 5 µM. Levels of 2HB were found to be stable in serum after three freeze-thaw cycles, and at ambient temperature and at a temperature of 4 °C for up to 24 h. Extracts derivatized under microwave irradiation were stable for up to 96 h. No differences were found in 2HB concentrations measured in serum or plasma EDTA samples. In summary, the method is useful for a rapid, precise and accurate quantification of 2HB in serum samples assessed for the evaluation of dysglycemia and *diabetes mellitus*.

## 1. Introduction

Type 2 diabetes mellitus (T2DM) is a metabolic disorder that affects millions of individuals worldwide and it is usually associated with prevalent comorbidities such as hypertension, hyperlipidemia or obesity. Also, serious complications may develop as a result of T2DM, such as retinopathy, nephropathy or cardiovascular disease, among others [1]. A key mechanism related to T2DM is insulin cellular resistance, which leads to hyperglycemia [2]. In addition, insulin resistance is also described as a common mechanism for different associated comorbidities such as obesity and metabolic associated fatty liver disease [3,4]. At present, the T2DM diagnosis criteria recommended by the clinical guidelines of the American Diabetes Association include a fasting plasma glucose ≥126 mg/dL, a 2 h plasma glucose ≥200 mg/dL during a 75 g oral glucose tolerance test or an HbA1c > 6.5% [5]. Although T2DM is a chronic disease with different therapeutic options available, a great endocrinological challenge in recent years has been the identification of subjects who are at risk of it and the prevention of its early development. Prediabetes, which is defined by plasma glucose concentrations higher than normal but below the defined threshold of diabetes, is related to an increased risk of T2DM and cardiovascular disease [5].

Untargeted and targeted metabolomics studies are effective strategies to identify biomarkers related to prediabetes, insulin resistance and T2DM development. In this line, several studies evidence the ability of plasma concentrations of 2-hydroxybutyrate (2HB) to be a marker of glycemic status and, interestingly, an early indicator of the future development of dysglycemia and T2DM [6,7,8,9,10,11,12,13,14,15,16]. Most of these studies use different analytical techniques including GC-MS, LC-MS, UHPLC-MS, time of flight-MS and/or nuclear magnetic resonance detection, which are programmed to cover a wide portion of the metabolome. However, there is a lack of well-validated methods specifically intended for the quantification of 2HB in human serum. In fact, GC-MS methods already validated for 2HB [17,18] were developed to cover the measurement of different analytes, which have the advantage of allowing simultaneous measurements of associated metabolites. However, they require the use of multi-step and long derivatization reactions to cover the correct detection of the different analytes. Therefore, the present study aimed to develop and validate a simple method based on a rapid microwave assisted derivatization (MAD), but one that is highly accurate and precise for the quantification of 2HB in human serum, which may be useful both in clinical and research laboratories for the evaluation of glycemic status and the risk of developing T2DM. 

## 2. Results and Discussion

### 2.1. Method Optimization

**Figure 1:**
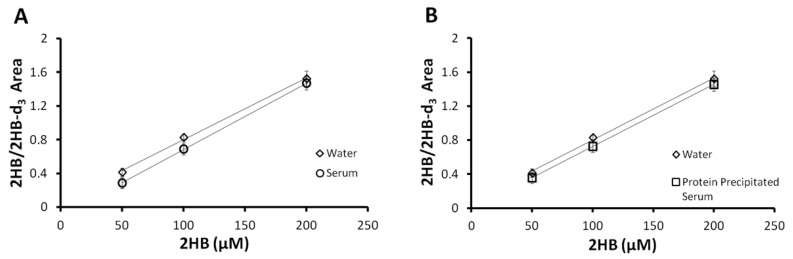
Matrix effect evaluation. (**A**) Three-point curves spiked in human serum compared with the respective curves spiked in water; (**B**) three-point curves spiked in protein precipitated human serum compared with the respective curves spiked in water.

The aim of this study was to develop an accurate and reproducible method for the quantification of 2HB in human serum which may be useful in clinical and research laboratories for the study of *diabetes mellitus* and the prevention and early diagnosis of T2DM. The method consisted of a simple liquid–liquid extraction of 300 µL of serum with ethyl acetate. Before extraction, serum samples were acidified with 90 µL of 5 M HCl to facilitate 2HB stability and detection. The main novelty of the study is that derivatization was reduced to a simple and very short incubation reaction with BSTFA:TMCS. The retention time was shorter than 4 min, which further facilitates the use of the method in routine applications. Standard curves were prepared in water to avoid the effect of endogenous 2HB that may be present in human serums. Although 2HB-d_3_ was used as the internal standard to compensate for any variations during sample processing, additional validation experiments were included to assess the suitability of the preparation of standards in water. Validation procedures which were performed to evaluate the differences in the recoveries of spiked 2HB between water and serum showed no significant differences in the slope coefficient (α) of 3-point 2HB-spiked curves prepared in water or serum (Figure 1A). Response factors (RF) were calculated as α spiked serum/α water or α spiked protein precipitated serum/α water, obtaining no significant differences. Thus, an RF was not necessary despite using a different matrix for calibration curves and clinical serum samples. These results support the parsimonious approach of not compensating for different matrices. Additionally, these results were no different from 3-point 2HB-spiked curves in protein precipitated serum before liquid–liquid extraction (Figure 1B), which was also a helpful indicator of the potential unnecessariness of protein precipitation before liquid–liquid extraction with ethyl acetate. Therefore, this step was not included in the extraction protocol of the method and was not performed in further validation steps except for the recovery assessment of 2HB in spiked samples. 

After extraction, to enhance the efficiency of gas chromatography and to improve the detectability and the selectivity of the analysis, polar metabolites are usually derivatized. Therefore, after solvent evaporation, 2HB was derivatized. In particular, MAD with BSTFA with 1% TMCS was evaluated for its readiness. In agreement with a similar MAD previous approach for the rapid derivatization of the structurally similar metabolite 2-hydroxylgutarate [19], successful silylation was achieved after only 2 min of microwave irradiation of standard extracts with BSTFA:TMCS 99:1, with a RF of 0.75 ± 0.1 compared to 1 h incubation at 60 °C. Therefore, the combination of BSTFA:TMCS 99:1 and MAD was chosen since this procedure satisfactorily achieved a simple and fast derivatization of 2HB.

**Figure 2:**
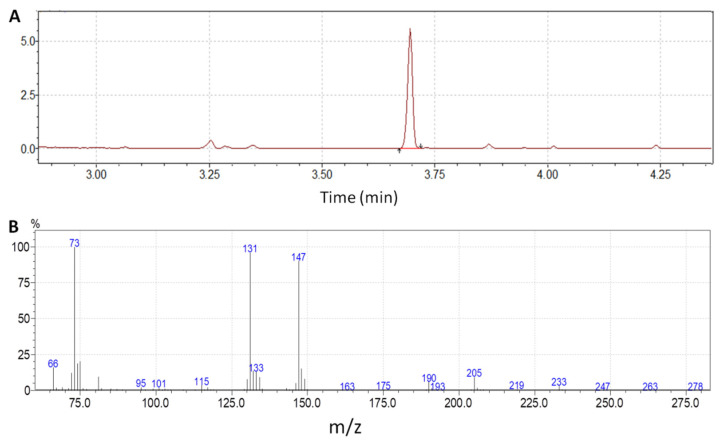
(**A**) Chromatogram of a serum sample (*m*/*z* 205); (**B**) mass spectrum of 2HB di-TMS.

After extraction and derivatization, the chromatographic conditions led to the profile shown in Figure 2. The retention time of 2HB was 3.7 min. The full scan spectrum for 2HB di-TMS is also shown in Figure 2. Synchronous selected ion monitoring (SIM)/scan acquisition was used to combine the achievement of low detection limits with highly specific measurements. 

### 2.2. Method Validation

Some methods for the determination of 2HB in serum can be found, with varying degrees of detail with respect to methodological description, in the scientific literature, especially in the context of the analysis of broad metabolic profiles. However, there are few validated methods for the determination of this metabolite in serum samples. Although mass spectrometry may be considered the gold standard method for the measurement of metabolites, quantitative methods based on mass spectrometry also require careful validation to provide objective evidence that the method is valid for the intended use and that it exhibits appropriate performance characteristics [20,21]. In our case, we performed a double validation of the method following the EMEA guideline [22], with two different internal standards, 2HB-d_3_ and 3HB-d_4_. This allowed us to detect the inappropriateness of using 3HB-d_4_ as the internal standard in our method since although the precision and accuracy of standards were good when using 3HB-d_4_ as an internal standard, the precision obtained in quality controls and the serum sample ranged from 10–65% and the accuracy of the serum sample was up to 180%, thus not meeting the validation requirements (Supplementary Table S1). Conversely, the use of 2HB-d_3_ as an internal standard greatly improved the accuracy of the method and met all the validation goals. These results show the need to ensure that the developed methods meet the analytical requirements for their intended use even if internal standards are structurally related. For example, quantification in many metabolomic studies is based on a few internal standards for different types of analytes, and confirmation of the obtained results may be necessary with a validated method.

Our method was linear for 2HB concentrations ranging from 5 to 200 µM, obtaining r values of 0.998 (mean) ± 0.004 (SD). Concentrations of calibration curve standards were established in this range to cover most of the expected results in diabetic and non-diabetic populations found in previous studies which show increased serum concentrations of 2HB in subjects with insulin resistance and/or T2DM in comparison with healthy individuals [7,9,17,23]. Despite the relatively wide range of the standard curve, the accuracy of calibration samples was above 85%, and the precision below 15%, as specified in the current guidelines for analysis [22]. The values of the inter-assay precision and the accuracy of the six calibrators are shown in Table 1.

The lowest calibrator (5 µM) was chosen for the LLOQ. The inter-day accuracy and inter-day precision of the LLOQ also met the validation requirement and were 100% and 8%, respectively (Table 1). The values of intra- and inter-assay precision and accuracy of two QC levels and the values of intra- and inter-assay precision of two serum samples also met the validation requirement and are summarized in Table 2.

Recoveries of added 2HB when analyzing three different spiked serums with two different amounts of 2HB (50 and 150 μM) ranged from 97–98%. These good recoveries were similar to those obtained when spiking the same amounts of 2HB to protein precipitates of the same serum samples (94–96%). No interfering signals were observed for 2HB and 2HB-d_3_ when analyzing different human serum samples. Similar ion ratios were observed between the quantification ions (*m/z* 205 for 2HB and *m/z* 208 for 2HB-d_3_) and the other two main ions (*m/z* 190 and *m/z* 233 for 2HB; *m/z* 193 and *m/z* 236 for 2HB-d_3_). Also, a carry-over effect was not observed after injecting blanks (cyclohexane) after the higher standard.

Few methods have been validated for 2HB measurements. Recently, Özkan et al. [18] developed and validated a method for the simultaneous determination of seven metabolites, which included 2HB. Also, Chou et al. [17] developed and validated a multi-analyte method to study isolated post-challenge diabetes. The differences and similarities between both of these methods and ours are displayed in Supplementary Table S2. In brief, all three are GC-MS based methods. Our method was focused specifically on 2HB measurements, whereas the other two methods measured several other analytes. Therefore, different internal standards were used in each method (myristic acid-d_27_, succinate-d_4_ and 2HB-d_3_). Özkan et al. used 100 µL of plasma, whereas we used a different type of sample (serum) and a higher volume of samples (300 µL). Both previous methods used protein precipitation with acetone or acetonitrile, whereas we used liquid–liquid extraction with ethyl acetate. In addition, our derivatization approach was quite different. Both the Özkan et al. and Chou et al. methods included a double derivatization with a combined incubation time of 115 min and 170 min, respectively. In comparison, our method avoids these long multi-step derivatization reactions required to detect the different analytes intended in these methods, and includes a MAD protocol that resulted in sTable 2HB-diTMS derivatives in a single reaction with a very short incubation time. Specifically, only a 2-min microwave irradiation was performed to achieve sTable 2HB-diTMS derivatives. Also, the specific optimization of our method for 2HB measurement resulted in a retention time of only 3.7 min, which was significantly shorter in comparison with the retention times, 7.5 min and 11.0 min, respectively, of the previously validated methods (Supplementary Table S2). Quantification ranges and the concentration values of 2HB obtained in the control subjects of each study are also displayed in Supplementary Table S2. As a limitation of our method validation, we did not perform a comparison of sample results between our method and those previously reported.

The stability of 2HB in serum stored at 4 °C (24 h), stored at room temperature (24 h) and after three freeze and thaw cycles was evaluated. The accuracy of both a low and a high serum concentration sample ranged from 99–104% in all these three different conditions (Table 3). These results are in agreement with those previously published in short-term and long-term stability evaluations of 2HB in serum and plasma human samples [17,18]. Moreover, as shown in Table 4, 2HB extracts in the autosampler of both QC samples and serum samples at room temperature were highly stable for at least 96 h. This result supports the efficacy of fast trimethylsilyl ester derivatization obtained by MAD.

### 2.3. Clinical Application

Finally, the validation included the measurement of 2HB in serum samples from individuals with high insulin and glucose, whose 2HB concentrations were compared with those obtained in serum samples of individuals with normal glucose and insulin values. Previous studies, mainly based on metabolomics, provide consistent evidence of the clinical value of 2HB measurements as a marker of glycemic status and, remarkably, of early prediction of progression to T2DM. Importantly, Goodman et al. [24] recently provided evidence that circulating 2HB levels and glucose tolerance can lie downstream of the hepatic cytosolic NADH/NAD+ ratio, and a more recent study suggests that 2HB is causally associated with T2DM and glycemic traits [25].

A comprehensive two-dimensional GC-TOF-MS study for biomarker discovery identified 2HB as a marker for T2DM in 2009 [6]. In a targeted metabolomics study using UHPLC-MS, 2HB was associated with insulin resistance and glucose intolerance and was found to be an independent predictor of dysglycemia or T2DM [7]. A metabolic approach based on GC-MS found 2HB to be also a biomarker for metabolic syndrome [8]. In an important study, Cobb et al. performed a targeted metabolomic analysis of 4053 subjects without diabetes at time 0 of an oral glucose tolerance test (OGTT). 2HB was found to be a selective biomarker of isolated impaired glucose tolerance and potentially useful in predictive models to identify subjects with impaired glucose tolerance without performing an OGTT [9]. In this regard, 2HB was included in a novel insulin resistance index to monitor changes in insulin sensitivity and glucose tolerance [26]. A combined targeted and untargeted metabolomics study found that 2HB predicted future progression to T2DM in a healthy, normoglycaemic population, years before the onset of T2DM [10]. Liu et al. [11] integrated metabolomic and genetic data and found that 2HB was associated with maternal insulin sensitivity before and 1 h after a glucose load. Other metabolomic studies also found higher levels of 2HB in patients with impaired fasting glucose and T2DM [12] and in obese T2DM vs. obese non-diabetic African-American women [13]. Using a non-targeted GC-MS and UHPLC-MS biochemical profiling conducted in nondiabetic subjects, random forest statistical analysis selected 2HB as the top-ranked molecule for separating insulin resistant from insulin sensitive subjects and also independently identified subjects with impaired fasting glycemia or impaired glucose tolerance [14]. A targeted metabolomics study by nuclear magnetic resonance found elevated fasting 2HB levels in adolescents with reduced insulin sensitivity, which predicted worsening glycemic control [15]. Finally, a cross-sectional study including patients at increased risk of diabetes found that basal 2HB levels predicted elevated 1 h glucose during OGTT, thus showing that 2HB measurements could provide a rapid, inexpensive screening tool for detecting early subclinical hyperglycemia, β-cell dysfunction and increased risk of diabetes [23]. 

**Figure 3:**
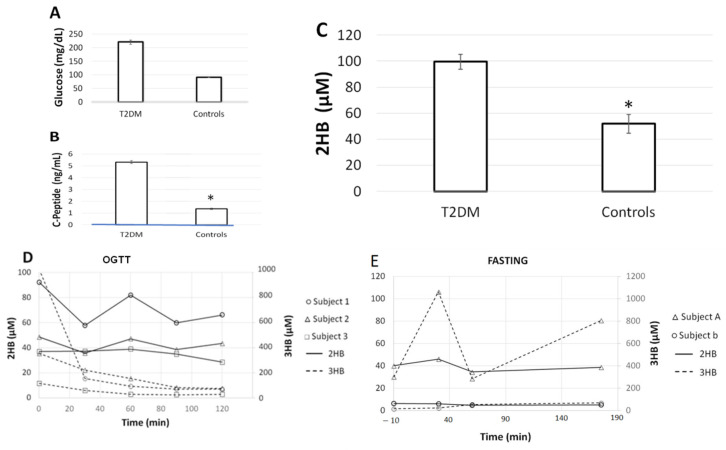
Basal serum levels (mean ± SEM) of (**A**) glucose, (**B**) C-Peptide and (**C**) 2-hydroxybutyrate (2HB). Serum levels of 2-hydroxybutyrate (2HB) and β-hydroxybutyrate (3HB) after (**D**) oral glucose tolerance test (OGTT) and (**E**) fasting up to 180 min. * *p* < 0.05, Student’s *t*-test.

In agreement with previous studies that obtained higher levels of 2HB in diabetic and pre-diabetic populations [6,7,9,12,16,23], we found that individuals with elevated glucose and C-peptide presented 2HB concentrations 2-fold higher than those obtained in individuals with normal glucose and C-peptide (99.4 ± 57.9 vs. 51.9 ± 35.9 µM; *p* < 0.05) (Figure 3A–C). In addition, 2HB levels measured in samples of OGTT and after 3 h fasting showed no evident changes over time (Figure 3D,E). This was in contrast with the glucose-induced reduction and fasting-induced elevation of β-hydroxybutyrate (3HB) (Figure 3D,E). Chromatographic resolution of 2HB and 3HB are shown in Supplementary Figure S1.

Finally, differences in levels of 2HB between serum and EDTA plasma samples from the same subjects were evaluated and non-significant differences were observed in a paired analysis (40 ± 28 vs. 39 ± 24 µM; *p* = 0.90; *n* = 10) (Supplementary Figure S2A). The serum–plasma mean differences were −3.2 ± 0.1%. To further evaluate the value of the method in quantifying plasma EDTA plasma samples, we evaluated the results of spiked 2HB in plasma EDTA and compared them with the 2HB standards prepared in water. As shown in Supplementary Figure S2B, recoveries of spiked EDTA in plasma samples were similar to those obtained in the calibration standards. Taken together, these results show that there is no effect with respect to blood extraction with EDTA in 2HB quantification in our method, which suggests that both serum and EDTA plasma may be used interchangeably.

## 3. Materials and Methods

### 3.1. Chemical Reagents

2-hydroxybutyric acid sodium salt, ethyl acetate, *N,O*-bis(trimethylsilyl)tri-fluoroacetamide:trimethylchlorosilane (BSTFA:TMCS; 99:1), barium hydroxide and zinc sulfate were obtained from Merck (Darmstad, Germany). Sodium 2-hydroxybutyrate-d_3_ (2HB-d_3_) was obtained from CDN Isotopes (Montreal, QC, Canada). Hydrochloric acid (37% (*w/w*) HCL) was purchased from Panreac (Barcelona, Spain). 3-hydroxybutyrate-d_4_ was obtained from Cayman Chemical (Ann Arbor, MI, USA). Ultrapure water was obtained using a Millipore Milli-Q purification system (Synergy, Merck Millipore, Burlington, MA, USA).

### 3.2. Instrumentation

GC-MS analyses were performed on a Shimadzu GCMS-QP2010 Ultra instrument (Kyoto, Japan) equipped with an electron impact source and a single quadrupole. Final sample extracts were injected into the GC-MS analyzer in splitless mode (valve open at 1 min). Chromatographic separation was achieved on a Sapines-5MS+ capillary column (30 m × 0.25 mm internal diameter × 0.25 μm film thickness) from Tecknokroma (Barcelona, Spain) using helium as the carrier gas at a constant velocity of 50 cm/s. The oven program temperature was started at 100 °C, maintained at this temperature for 2 min, elevated at 15 °C min^−1^ to 115 °C, then increased at 80 °C min^−1^ until 300 °C and finally maintained for 13 min at 300 °C. One microliter was injected into the chromatograph. The total run time was 18 min. Four pre- and post-injection washes (8 µL each) were realized with cyclohexane. The ion source and transfer line temperatures were set to 250 °C and 280 °C, respectively. A mass detector operated in synchronous selected ion monitoring (SIM) mode (*m/z* 190, 193, 205, 208, 233, 236) using dwell times of 150 ms and scan mode ranging from *m/z* 65 to *m/z* 280 using a dwell time of 30 ms. For quantitative analysis, we selected ions *m/z* 205 and *m/z* 208 for 2HB and 2HB-d_3_, respectively, to maximize specificity, and identification was also achieved by the comparison of GC retention times with reference standards. The specific ions monitored were selected based on the fragmentation pattern of the 2HB standard (Figure 2B), which was coincident with that of the National Institute and Technology (NIST) library [27]. Although higher abundant ions (*m/z* 73, *m/z* 131, *m/z* 147) than those selected were present in the mass spectrum, these ions are also commonly observed in the mass fragmentation pattern of other organic acids. Therefore, we selected ions with a higher mass to charge ratio to maximize the selectivity of the method.

### 3.3. Preparation of Stock Solutions, Working Solutions, Calibrator and Quality Control Samples

Calibration curves were prepared in water solutions as a 2HB free surrogate matrix due to the presence of 2HB endogenously in the human serum. Recovery assessments of spiked 2HB were used to evaluate the differences in the analytical response between both matrixes. A stock solution of 2HB was prepared at a concentration of 20 mM in water and stored at −20 °C. Working solutions were prepared by diluting the stock solution 1:20 in water to final concentrations of 1000 µM. Six-point calibration curves (5, 10, 50, 100, 150, 200 µM) were prepared by diluting 2HB working solution in water. Also, two-point quality controls (QC) (30 and 125 µM) were prepared by diluting 2HB working solutions in water. 

### 3.4. Sample Preparation

For the measurements of 2HB, 30 μL of 2HB-d_3_ (1 mM) were added as an internal standard to 300 μL of calibrators, QC and serum samples. All of them were then acidified with 90 μL of 5 M HCl, and 4 mL of ethyl acetate was added for liquid–liquid extraction. After centrifugation at 2500× *g* for 10 min, the organic phase was transferred and evaporated under nitrogen at 37 °C. Trimethylsilyl ester derivatives were formed by derivatization of extracts with 80 μL of BSTFA:TMCS 99:1 through 2-min microwave irradiation at 800 W. The selection of these conditions were based on their previous use on the successful microwave assisted sylilation of 2-hydroxyglutarate [19].

### 3.5. Microwave-Assisted Derivatization

Derivatization yields achieved when performing the silylation under thermal block or microwave irradiation were compared. When conducting the classical heating approach (thermal block), trimethylsilyl esters were formed by adding 80 μL of BSTFA:TMCS 99:1 to the extracts and heating for 60 min at 60 °C. For MAD, 80 μL of BSTFA:TMCS 99:1 were added to the final extracts and silylation was performed in a domestic microwave (SpeedyGrill, Taurus) at 800 W for 2 min. The relative response factors (RF) for 2HB were calculated by comparing the peak areas obtained by microwave assisted energy irradiation (MAD) with those obtained by thermal block heating derivatization.

### 3.6. Method Validation

#### 3.6.1. Linearity of Calibration Curves

For the evaluation of linearity, complete calibration curves over a range of between 5 and 200 µM using six concentrations (5, 10, 50, 100, 150 and 200 µM) were analyzed on five different days. Weighted linear regression was used to plot the peak area ratio (2HB to 2HB-d_3_) versus the corresponding 2HB concentration. Slope, y-intercept and correlation coefficient were calculated for each standard curve. A value of r ≤ 0.99 was required to pass this validation step. The precision and accuracy versus the nominal concentration of the six calibrators levels were also calculated. Back-calculated concentrations were acceptable when within ±15% of the nominal values except for the limit of quantification, for which a ±20% was considered [22].

#### 3.6.2. Matrix Effect Evaluation

The analytical responses of spiked 2HB in water or human serum were assessed to ensure that the calibration curve prepared in water calibrators could be used to quantify clinical human serum samples. The slope coefficient (α) of 3-point curves 2HB-spiked in serum from two different sources was compared with their respective standard curves 2HB-spiked in water. Response factors (RF) were calculated as α_serum_/α_water_. Both approaches were evaluated by comparing the back-calculated concentrations of spiked serum samples with and without RF correction to calculate the sum of absolute values of the relative residuals (relative residual = 100 × (C_serum_ − C _nominal_)/C_nominal_). For comparison, the same procedure was also performed with spiked protein precipitated serums (300 μL) with zinc sulfate and barium hydroxide, which were extracted using ethyl acetate after protein precipitation.

#### 3.6.3. Accuracy and Precision

The accuracy and the precision of the method were assessed by the back-calculated results of multiple measurements of the two QC (30 and 125 µM) and two serum samples. The accuracy and precision of the lower limit of quantification (LLOQ) and the upper limit of quantification (ULOQ) were also assessed and they were set at the lowest (5 µM) and highest (200 µM) calibration standard values, respectively. For the assessment of intra-day accuracy and precision, five replicates were analyzed for each concentration on the same day. Inter-day accuracy and precision were calculated on five separate days. To pass the accuracy test, the mean obtained values should be within 100 ± 15% of the theoretical value. Accuracy was determined as the difference between the calculated concentrations of 2HB and the theoretical concentration following the formula (C_obtained_ − C_theoretical_)/(C_theoretcal_). Precision at each concentration level was expressed as relative standard deviation (%RSD), which should not exceed 15%. In the case of the LLOQ, accuracy should stand within 100 ± 20% and precision should be lower than 20% [22].

#### 3.6.4. Recovery, Selectivity and Carry Over

The recovery of added 2HB was assessed by comparing the obtained and expected concentrations in the same serum samples used in the matrix effect evaluation with the formula Recovery (%) = (C_observed_ − C_expected_)/C_spiked_. Concentration result values were obtained using complete external standard curves. The selectivity was investigated by performing the analysis of 30 different serum samples from different individuals and was indicated by the absence of endogenous interferences at the retention times of 2HB and 2HB-d_3_. The evaluation of the carryover was performed by injecting 1 μL of cyclohexane immediately after the injection of a standard with ULOQ concentration (200 µM) on three different occasions.

#### 3.6.5. Stability of Microwave Assisted Derivatization and Stability of Serum Samples

The stability on the autosampler of extracts derivatized by MAD was evaluated by reinjecting two QC sample extracts and two human serum sample extracts stored inside the autosampler at ambient temperature after 24, 48 and 96 h. The stability of 2HB in serum samples was evaluated by reanalyzing in triplicate two samples that were stored under three different conditions: three freeze-thaw cycles, room temperature for 24 h and 4 °C for 24 h. 

### 3.7. Method Applicability

The concentration of 2HB was analyzed on remnants of serum samples of healthy subjects (*n* = 10) and subjects with high glucose and insulin (*n* = 10). Also, remnants of serum samples were used to measure 2HB in oral glucose tolerance tests (*n* = 3) and after fasting (*n* = 2). Serum levels of 3HB were also measured in these samples following a previously described method [28]. Serum glucose and C-peptide were measured on Atellica and Immulite instruments, respectively (Siemens Healthineers, Tarrytown, NY, USA). In addition, 2HB was measured in 10 serum and their corresponding plasma EDTA samples from the same blood extraction in order to evaluate the differences between both specimens.

### 3.8. Statistics

Statistical analyses were performed with the GraphPad Prism 6 (GraphPad Prism Software Inc., San Diego, CA, USA) and RStudio Team (2020). RStudio: Integrated Development for R. RStudio, PBC, Boston, MA, USA. This investigation has complied with the World Medical Association Declaration of Helsinki regarding the ethical conduct of research.

## 4. Conclusions

A routine GC-MS method was developed and validated for the quantitative measurement of 2HB in human serum. The method comprises a simple liquid–liquid extraction followed by a fast one step derivatization achieved through 2 min of microwave irradiation. The intra- and inter-assay accuracy and precision are within the EMEA guidelines validation criteria. The intra- and inter-day imprecision of QC and serum samples were <8%, the accuracy ranged from 96–101%, the stability of the derivatized extracts ranged from 89–109% and the LLOQ was 5 µM. 2HB was found to be stable in serum under different storage conditions, and the values found in serum and EDTA plasma samples showed comparable concentration results. In summary, we demonstrated that our GC-MS based 2HB quantification can be performed in a robust manner for the evaluation of alterations in glucose metabolism and T2DM in research and clinical laboratories.

## Figures and Tables

**Table 1:** Inter-day precision and accuracy values of the calibration curve standards (*n* = 5).

Standard	2HB (µM)	Precision (%)	Accuracy (%)
Std 1	5	8.0	100.3
Std 2	10	3.6	97.3
Std 3	50	2.3	102.6
Std 4	100	2.4	103.8
Std 5	150	2.3	98.5
Std 6	200	1.9	99.1

**Table 2:** Intra-day (*n* = 5) and inter-day (*n* = 5) precision and accuracy values of 2HB measurements in quality control and serum samples.

	2HB (µM)	Precision (%)		Accuracy (%)	
		Intra-Day	Inter-Day	Intra-Day	Inter-Day
QC1	30	1.7	2.4	96.4	100.0
QC2	125	1.1	0.7	100.9	100.9
Serum 1	34.5 *	7.1	3.0	NA	NA
Serum 2	80.3 *	2.6	4.9	97.3 ^+^	100.1 ^+^

QC: quality control. * Obtained mean value. ^+^ Calculated as the recovery of 2HB spiked to serum.

**Table 3:** Stability of 2HB in human serums expressed as accuracy (*n* = 3).

	Concentration	Accuracy (%)		
	Mean (µM)	24 h (25 °C)	24 h (4 °C)	F&T ^1^ (3 Cycles)
Serum 1	33.3	99 ± 3	104 ± 1	101 ± 1
Serum 2	80.2	103 ± 6	103 ± 7	101 ± 7

^1^ F&T: freeze-thaw.

**Table 4:** Stability in the autosampler at ambient temperature expressed as accuracy values of 2HB after 24, 48 and 96 h.

	Concentration	Accuracy (%)		
	(µM)	24 h	48 h	96 h
QC 1	29.4	103.3	106.5	108.6
QC 2	125,6	100.0	103.3	102.7
Serum 1	33.3	88.9	89.5	94.4
Serum 2	80.2	101.5	101.0	106.9

QC: Quality control.

## Data Availability

Not applicable.

## Supplementary Materials

The following supporting information can be downloaded at: https://www.mdpi.com/article/10.3390/molecules27061889/s1. Table S1: Precision and accuracy of six calibrators, two quality controls and two serum samples using 3HB-d_4_ as internal standard; Table S2: Comparison of the characteristics of our GC-MS method with those of two previously validated methods for 2HB measurements in serum or plasma; Figure S1: Chromatogram of a serum sample showing chromatographic resolution of 2-hydroxybutyrate (2HB) and 3-hydroxybutyrate (3HB); Figure S2: (**a**) Comparison of the results obtained in EDTA plasma and serum samples from the same blood extraction (*n* = 10). (**b**) Three-point curves spiked in human EDTA plasma compared with the respective curves spiked in water.
