# Supplementary material for: Validation of a Microwave-Assisted Derivatization Gas Chromatography-Mass Spectrometry Method for the Quantification of 2-Hydroxybutyrate in Human Serum as an Early Marker of Diabetes Mellitus
Source: Molecules. 2022 Mar 15;27(6):1889. doi: 10.3390/molecules27061889 (PMC8950062; doi:10.3390/molecules27061889)
Supplement: Supplementary file 1 [file molecules-27-01889-s001.zip › molecules-1613067-supplementary.pdf]

molecules

MDPI

Table S1. Precision and accuracy of 6 calibrators, two quality controls and two serum samples using 3HB-d_4 as internal standard.

	2HB (µM)	Precision (%)	Accuracy (%)
Std 1	5	14	88
Std 2	10	12	81
Std 3	50	13	95
Std 4	100	8	105
Std 5	150	5	99
Std 6	200	4	100
QC 1	30	26	100
QC 2	125	10	103
Serum 1	86*	65	NA
Serum 2	244*	61	180+

Std: Standard. QC: Quality Control.

* Obtained mean value. + Calculated as the recovery of 2HB spiked to serum 1.

Table S2. Comparison of the characteristics of our GC-MS method with those of two previously validated methods for 2HB measurements in serum or plasma.

	**Özkan *et al.* [23]**	**Chou *et al.* [22]**	**Our Method**
Method	GC-MS	GC-MS	GC-MS
Intended use	pregnant women (screening Down Syndrome)	Isolated post-challenge diabetes	Dysglycemia and T2DM
Sample type	Plasma	Serum	Serum
Sample volume	100 µL	100 µL	300 µL
Internal standard	Myristic acid-d_{27}	Succinate –d_4	2HB-d_3
Sample extraction	Protein precipitation (acetonitrile)	Protein precipitation (acetone)	Liquid:liquid (ethyl acetate)
Derivatization	MEOX pyridine (30ºC, 90 min) & MSTFA+1%TMCS (60ºC, 25 min)	MEOX pyridine (37ºC, 90 min) & MSTFA (60ºC 10 min) & 70 min at 50ºC	BSTFA +1%TMCS (MAD, 2 min)
Retention time (min)	7.5	11.0	3.7
Limit of detection (µM)	2.4	0.96	NA
Limit of quantification (µM)	8.0	NA	5.0
Calibration range (µM)	8-480	NA-865	5-200
Values in control subjects (µM)	10 *	87±29	52±36

BSTFA+1%TMCS: O-Bis(trimethylsilyl)trifluoroacetamide +1 % trimethylchlorosilane. MAD: micowave assisted derivatization. NA: Not available. MEOX: methoxyamine hydrochloride. MSTFA+1%TMCS: N-methyl-N-trimethylsilyl-trifluoroacetamide+1 % trimethylchlorosilane. NA: not available. T2DM: type 2 diabetes mellitus * Mean concentration value of 11 to 15-week pregnant women (estimated from figure 3 of Reference 23).

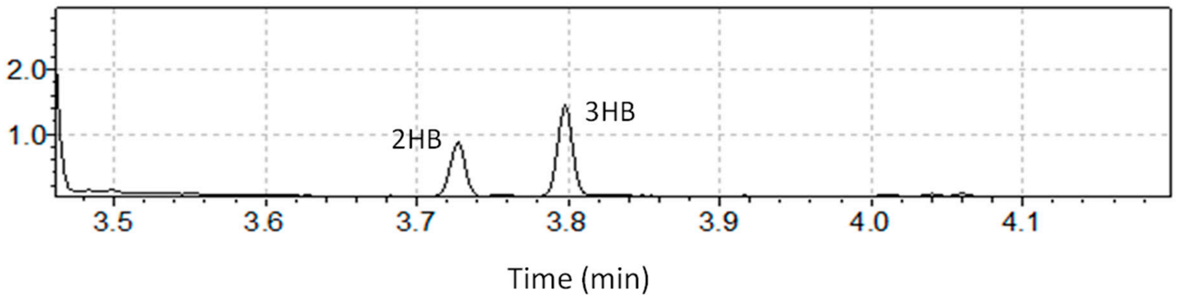

Figure S1. Chromatogram of a serum sample showing chromatographic resolution of 2-hydroxybutyrate (2HB) and 3-hydroxybutyrate (3HB).

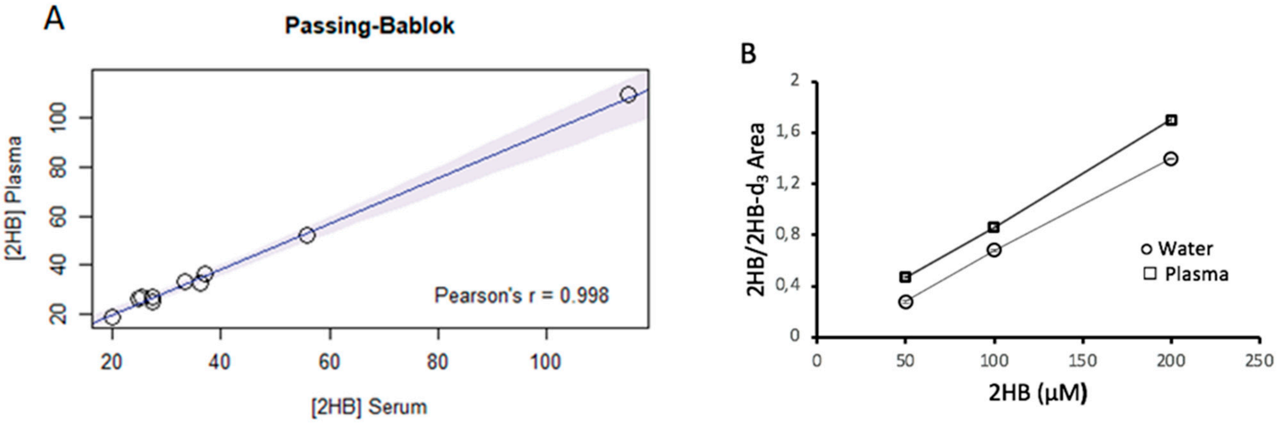

Figure S2. (a) Comparison of the results obtained in EDTA plasma and serum samples from the same blood extraction (n=10). **(b)** Three-point curves spiked in human EDTA plasma compared with the respective curves spiked in water.
